# Attenuated conflict self-referential information facilitating conflict resolution

**DOI:** 10.1038/s41539-024-00256-4

**Published:** 2024-07-19

**Authors:** Zhifang Li, Jing Wang, Yongqiang Chen, Qing Li, Shouhang Yin, Antao Chen

**Affiliations:** 1 School of Psychology, Research Center for Exercise and Brain Science, Shanghai University of Sport, Shanghai, 200438 China; 2 Key Laboratory of Cognition and Personality of Ministry of Education, Faculty of Psychology, Southwest University, Chongqing, 400715 China; 3 School of Psychology and Cognitive Science, East China Normal University, Shanghai, 200062 China; 4 School of Psychology, Liaoning Normal University, Dalian, 116029 China

**Keywords:** Human behaviour, Human behaviour

## Abstract

Self-referential information can reduce the congruency effect by acting as a signal to enhance cognitive control. However, it cannot be denied that self-referential information can attract and hold attention. To investigate this issue, the study used a revised Stroop task and recorded behavioral and electrophysiological data from thirty-three participants. We combined event-related potential (ERP) and multivariate pattern analysis (MVPA) to examine the neural correlates of self-referential processing and conflict processing. In the behavioral results, self-referential information reduced the congruency effect. Specifically, self-reference stimuli elicited smaller N2 amplitude than non-self-reference stimuli, indicating that self-referential information was promptly identified and reduced top-down cognitive resource consumption. Self-referential information could be reliably decoded from ERP signals in the early-to-mid stage. Moreover, self-reference conditions exhibited earlier congruency decoding than non-self-reference conditions, facilitating conflict monitoring. In the late stage, under the incongruent condition, self-reference stimuli elicited smaller sustained potential amplitude than non-self-reference stimuli, indicating that cognitive control in the self-reference condition required fewer cognitive resources for conflict resolution. Together, these findings revealed that self-referential information was identified and facilitated conflict monitoring, leading to more effective conflict resolution.

## Introduction

Cognitive control enables goal-guided behavior and shields from interference^1^. One common paradigm used to investigate cognitive control is the conflict task, such as the Stroop task^2^, where participants are required to name the color of a word (task-relevant dimensions) while ignoring its meaning (task-irrelevant dimensions). Typically, they responded slower in incongruent trials (word meaning different from color) than in congruent trials (word meaning same as color). This difference in reaction time between incongruent and congruent trials is referred to as the congruency effect (CE). Dignath et al.^3^ found that self-referential primes reduced the CE. They proposed that self-referential information can act as a signal to enhance cognitive control, but cannot deny the possibility that self-referential information may attract and hold attention. However, their investigation was currently limited to the behavioral level, and the available evidence was insufficient. Therefore, the current study aims to employ both behavioral and neural methods (electroencephalography, EEG) to test our hypotheses and propose a specific time course.

Self-referential information exhibits a self-prioritization effect (SPE) during cognitive processing, such as memory^4,5^ and attention capture^6,7^. People usually process information about themselves more quickly than information about others^8^. This implies that self-referential information has a processing advantage over other-referential information. It’s noteworthy that this processing advantage also appears to extend to conflict processing. Dignath et al.^3^ found that self-referential priming can reduce the CE. Their control hypothesis suggests that self-referential information bolsters cognitive control to protect against interference from irrelevant stimuli. Despite this, several limitations should be considered. First, the control account is mainly based on this single study, relying on conjecture drawn from behavioral results; Second, priming might induce an effect akin to a negative Stimulus Onset Asynchrony (SOA), particularly evident in Experiment 2 of Dignath et al.^3^. Even in the absence of self-referential information, priming with a negative SOA could still influence the CE^9–11^. Therefore, we conducted a study to test the validity of the control hypothesis by eliminating priming^12^. Our study revealed that in event-based designs, self-referential information didn’t reduce the CE; nonetheless; in block designs, self-referential information can reduce the CE. This discrepancy may stem from continuous exposure to self-referential information in blocks, which may produce effects similar to those of self-referential priming. Previous investigations have relied solely on behavioral outcomes for inference, without exploring the neural mechanisms underlying the impact of self-referential information on conflict processing. Thus, our study employed the block design incorporating self-referential information within the modified Stroop task, as utilized by Li et al.^12^, to examine the temporal processes underlying this phenomenon.

EEG technology has the characteristic of high temporal resolution. Event-related potential (ERP) methods provide important evidence on self-referential information in which N2 amplitude^13–17^. The N2 is believed to signify early higher-order operations related to stimulus discrimination and categorization^18^. Self-referential stimuli can be more readily retrieved with reduced top-down cognitive resource consumption. The N450 amplitude is an indicator of conflict monitoring^19–21^. The sustained potential (SP) amplitude is related to conflict resolution and attention resource allocation^22–25^.

Multivariate pattern analysis (MVPA) is a method that considers the relationships between multiple variables (channels in EEG) instead of treating them as independent^26^. Decoding, an application of MVPA, uses machine learning classifiers to identify the task or stimulus from patterns of brain activity^27,28^. By repeating the classification at different time points, we can examine the temporal course of information processing in the brain. Above-chance decoding occurs when the decoding accuracy is significantly above the chance level, indicating that the machine learning model successfully discriminates between different brain activity patterns associated with experimental conditions^29–31^. Weight projection is a method to estimate the contribution of different brain regions (channels) to the classification performance^32,33^. Importantly, MVPA is relatively sensitive to decoding the time course of self-referential information and congruency^29,32^, and supports further examination of decoding performance by weight projection^26^. Therefore, ERP and MVPA methods may elucidate the temporal dynamics of how self-referential information influences conflict processing.

The present study investigated the effects of self-referential information and congruency on behavioral and EEG measures in a revised Stroop task. Our central hypothesis was that self-referential information was identified^16,34^ and facilitated conflict monitoring. This would reduce the cognitive resources needed to resolve conflicts in the late stage^3,12^. Consequently, N2 amplitude and above-chance decoding should differ between self-reference and non-self-reference conditions since self-referential processing occurred in the early stage. Conflict should be detected earlier under self-reference conditions, as indicated by an earlier N450 amplitude and earlier above-chance decoding. Furthermore, a divergence between self-reference and non-self-reference should be observed in SP amplitude and above-chance decoding, as conflict resolution recruited fewer cognitive resources under self-reference conditions.

## Results

### Behavioral results

Initially, this study excluded 0.9% of the first trial of each block, 5.9% of response error trials, 5.8% of post-error trials from further analyses, and 1.0% of trials with RTs exceeding 3 SDs above or below the mean RT.

**Fig. 1:**
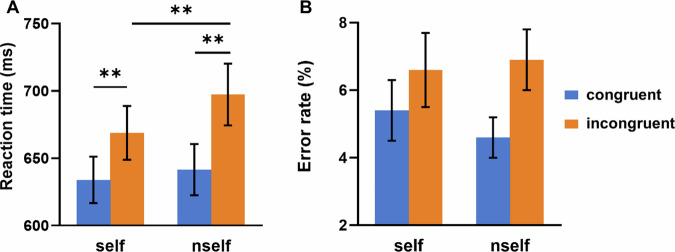
Behavioral results in the modified Stroop task. The overall reaction time (**A**) and error rate (**B**) on trials involving self-referential information and congruency condition. Error bars indicate the standard error of the mean. *p < 0.05. **p < 0.01. ***p < 0.001.

Behavioral results are shown in Fig. 1.

As for error rates, a repeated-measures ANOVA was conducted, considering both congruency (congruent vs. incongruent) and self-referential information (the self-reference condition “my” vs. the non-self-reference condition “the”). The results did not show a significant interaction between these factors, *F*(1, 32) = 2.84, *p* = 0.102, η_p_^2^ = 0.08. The main effect of congruency was significant, *F*(1, 32) = 19.01, *p* < 0.001, η_p_^2^ = 0.37, indicating that the congruent condition exhibited lower error rates (5.0%) compared to the incongruent condition (6.8%).

Regarding RT, a repeated measures ANOVA with 2 (congruency: congruent vs incongruent) × 2 (self-referential information: the self-reference condition “my” vs the non-self-reference condition “the”) yielded a significant interaction, *F*(1, 32) = 12.19, *p* = 0.001, η_p_^2^ = 0.28. Specifically, self-reference condition (34.88 ms) reduced the CE compared to the non-self-reference condition (55.77 ms). Subsequently, simple effects analyses (refer to the effect of an independent variable on a dependent variable at a particular level of another independent variable) were employed to deconstruct the interaction between the two factors, and the results are presented in Fig. 1A. In the incongruent condition, there was a significant difference between self-reference and non-self-reference conditions, with self-reference resulting in faster RTs (668.84 ms) than non-self-reference condition (697.36 ms), *F*(1,32) = 18.51, *p* < 0.001. In the self-reference condition, there was a significant difference between congruent and incongruent conditions, with congruent trials resulting in faster RTs (633.97 ms) than incongruent trials (668.84 ms), *F*(1,32) = 38.20, *p* < 0.001. Similarly, in the non-self-reference condition, there was a significant difference between congruent and incongruent conditions, with congruent trials resulting in faster RTs (641.58 ms) than incongruent trials (697.36 ms), *F*(1,32) = 79.06, *p* < 0.001.

### ERP results

**Fig. 2:**
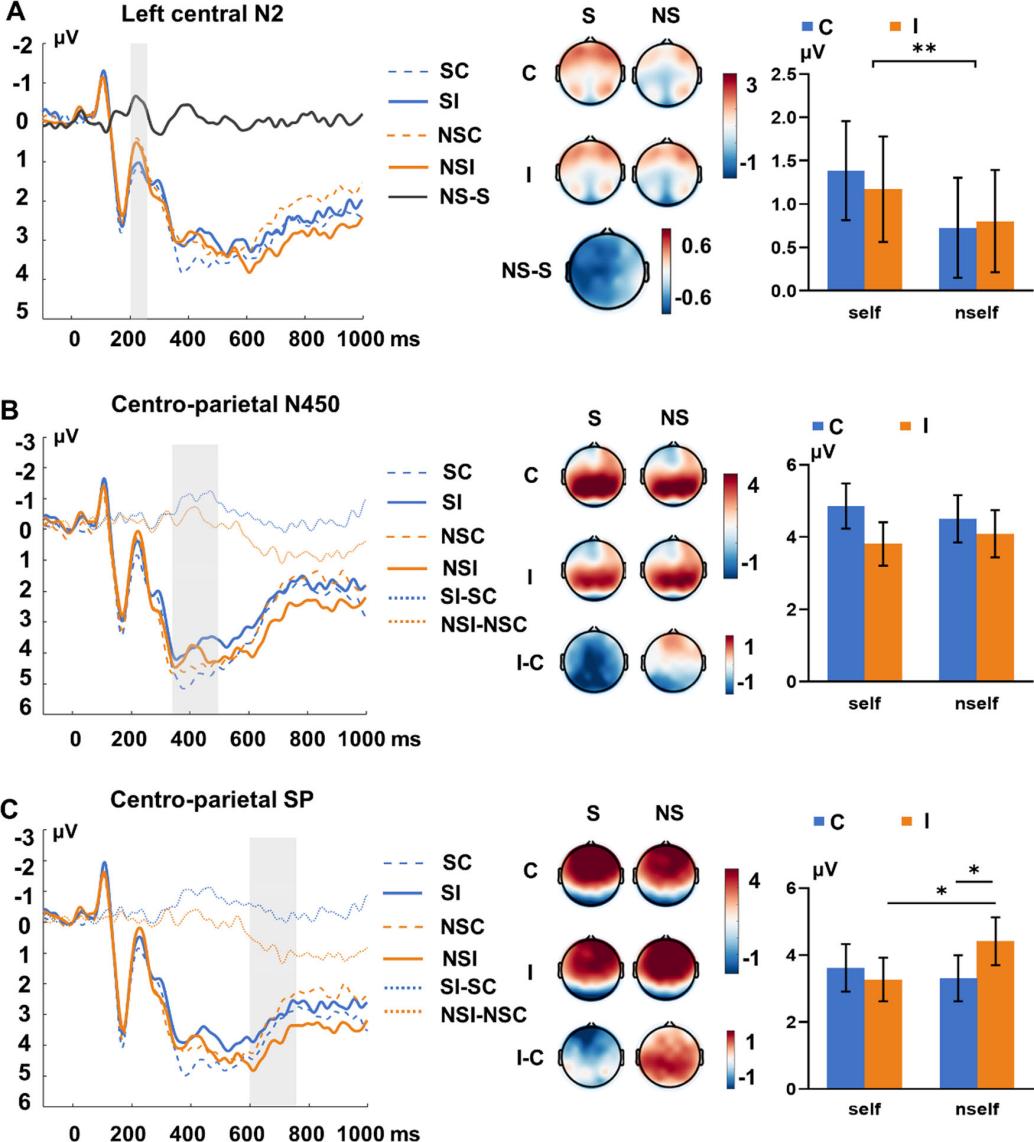
ERP results in the modified Stroop task. The waveform, topography, and amplitude of N2 (**A**), N450 (**B**), and SP (**C**) for self-referential information and congruency condition. The shaded regions represent the time windows for N2, N450, and SP, respectively. The topography distributions represent the average amplitude in time windows. Error bars indicate the standard error of the mean. *p < 0.05. **p < 0.01. ***p < 0.001. Note: S = self-reference condition, NS = non-self-reference condition, C congruent, I incongruent, SC self-reference and congruent condition, SI self-reference and incongruent condition, NSC non-self-reference and congruent condition, NSI non-self-reference and incongruent condition.

ERP results are illustrated in Fig. 2.

#### N2

The ANOVA analysis for the stimuli-locked N2 amplitude showed that the interaction between the congruency condition and self-reference condition was not significant, *F*(1, 32) = 1.359, *p* = 0.252, η_p_^2^ = 0.04. The main effect of congruency was not significant, *F* < 1. The main effect of self-referential information was significant, *F*(1, 32) = 7.16, *p* = 0.012, η_p_^2^ = 0.18, indicating that the N2 amplitude evoked by non-self-reference condition (0.76, *SE* = 0.57) was significantly greater (more negative) than that evoked by self-reference condition (1.28, *SE* = 0.58), as shown in Fig. 2A.

#### N450

The ANOVA analysis for the stimuli-locked N450 amplitude showed that the interaction between congruency and self-referential stimuli type was not significant, *F*(1, 32) = 3.84, *p* = 0.059, η_p_^2^ = 0.10. The main effect of congruency was significant, *F*(1, 32) = 7.46, *p* = 0.010, η_p_^2^ = 0.19, indicating that the N450 amplitude evoked by incongruent condition (3.94, *SE* = 0.60) was significantly greater (more negative) than that evoked by congruent condition (4.68, *SE* = 0.62), as depicted in Fig. 2B. The main effect of self-referential information was not significant, *F* < 1.

#### SP

The ANOVA analysis for the stimuli-locked SP amplitude showed a significant interaction between congruency and self-referential stimuli type, *F*(1, 32) = 5.16, *p* = 0.030, η_p_^2^ = 0.14. The main effect of congruency was not significant, *F* < 1. The main effect of self-referential information was not significant, *F*(1, 32) = 1.09, *p* = 0.304, η_p_^2^ = 0.03. To further explore the interaction between self-referential information and congruency, simple effects analyses were conducted, and the results are presented in Fig. 2C. In the non-self-reference condition, there was a significant difference between congruent and incongruent conditions, *F*(1, 32) = 5.51, *p* = 0.025, indicating that the SP amplitude evoked by incongruent condition (4.15, *SE* = 0.72) was significantly greater (more positive) than that evoked by congruent condition (3.31, *SE* = 0.69). In the incongruent condition, there was a significant difference between self-reference and non-self-reference conditions, *F*(1, 32) = 4.55, *p* = 0.041, suggesting that the SP amplitude evoked by non-self-reference condition (4.15, *SE* = 0.72) was greater (more positive) than that evoked by self-reference condition (3.27, *SE* = 0.65).

### MVPA results

**Fig. 3:**
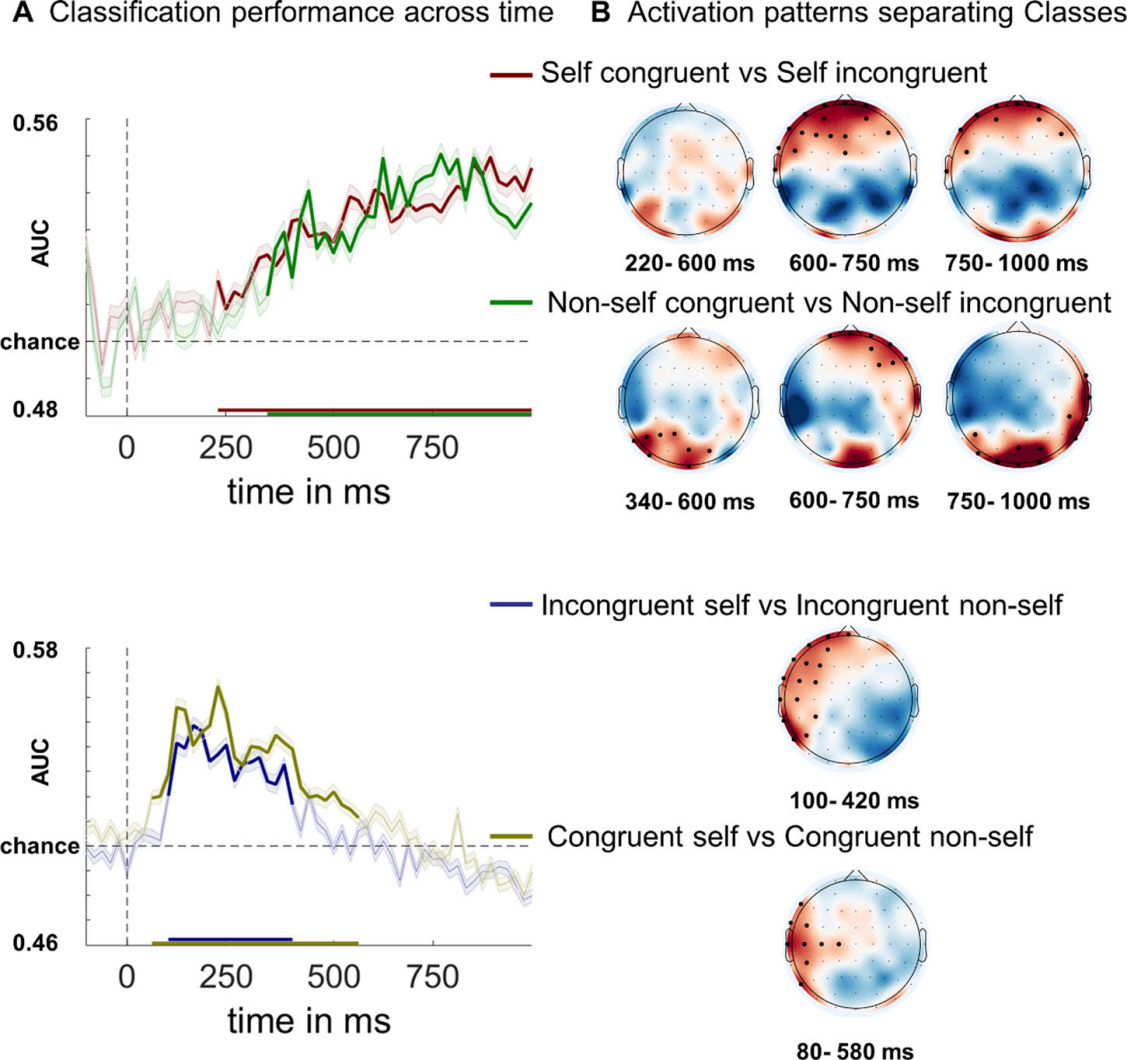
MVPA results in the modified Stroop task. **A** The classification accuracy for the four datasets, with shicker lines indicating the time windows of significant classification performance (*p* < 0.001, cluster-corrected). **B** The maps of forward transformation classifier weights averaged over the time windows of significant classification performance.

MVPA results are displayed in Fig. 3.

To determine the temporally extended pattern of neural activity after self-referential involvement in conflict tasks, linear discriminant classifiers were trained at each time point to perform the following: (1) distinguish between self-reference-congruent and self-reference-incongruent; (2) distinguish between non-self-reference-congruent and non-self-reference-incongruent; (3) distinguish between incongruent-self-reference and incongruent-non-self-reference; (4) distinguish between congruent-self-reference and incongruent-non-self-reference.

As depicted in Fig. 3A, regarding self-reference-congruent and self-reference-incongruent comparison, MVPA revealed a significant above-chance (AUC > 0.5) difference between the two classes from 220 to 1000 ms after stimulus onset (*p* < 0.001, cluster-corrected, both-sided). Similarly, for non-self-reference-congruent and non-self-reference-incongruent comparison, MVPA revealed a significant above-chance difference between the two classes from 340 to 1000 ms after stimulus onset (*p* < 0.001, cluster-corrected, both-sided). As depicted in Fig. 3B, with respect to incongruent-self-reference and incongruent-non-self-reference, MVPA revealed a significant above-chance difference between the two classes from 100 to 420 ms after stimulus onset (*p* < 0.001, cluster-corrected, right-sided). For congruent-self-reference and congruent-non-self-reference, MVPA revealed a significant above-chance difference between classes of congruent-self-reference and incongruent-non-self-reference from 80 to 580 ms after stimulus onset (*p* < 0.001, cluster-corrected, right-sided). These results suggest that, for self-referential information, the two types of self-referential information (either incongruent or congruent) could be differentiated in the early-to-mid stage after stimuli presentation. On the other hand, for congruency, the two types of congruencies (either self-reference or non-self-reference) could be differentiated in the mid-to-late stage after stimuli presentation. Importantly, the self-reference condition could classify congruent and incongruent conditions earlier than the non-self-reference condition.

During the time windows of significant classification performance, the topographical patterns of forward transformation classifier weights with high spatial resolution revealed that the spatial activity patterns differed across different datasets. It’s noteworthy that both self-reference-congruent/incongruent comparisons and non-self-reference-congruent/incongruent comparisons displayed clearly significant clusters after cluster-based permutation tests, ranging from 700 to 1000 ms. Interestingly, the significant cluster for self-reference comparisons were localized in the anterior region, while that of non-self-reference comparisons were localized in the posterior region. Additionally, both congruent self-reference/non-self-reference comparisons (80 to 580 ms) and incongruent self-reference/non-self-reference comparisons (100 to 420 ms) displayed significant clusters in the left anterior region, as illustrated in Fig. 3B.

## Discussion

We used a modified Stroop task and EEG to examine how self-referential information impacts conflict processing. Behaviorally, self-referential information reduced the congruency effect (CE) replicating a previous study^3,12^. ERP results showed that the self-reference condition elicited a smaller N2 amplitude than the non-self-reference condition in the early processing stage. In the conflict monitoring stage, the incongruent condition elicited larger N450 amplitude than the congruent condition. Furthermore, under the incongruent condition, the self-reference condition elicited a smaller SP amplitude than the non-self-reference condition in the conflict resolution stage. MVPA revealed that the self-reference condition discriminated against conflict earlier than the non-self-reference condition. Additionally, both self-reference and non-self-reference conditions were distinguished in the early-to-mid stage after stimulus onset.

In line with the research paradigm of Experiment 2 by Dignath et al.^3^, we removed the priming and implemented a block design of self-referential information. We still found that self-referential information reduced the CE with block-wise design^12^. Both priming and block-wise designs seem to enhance the modulation of attentional resources effectively by self-referential information. A notable similarity between these designs may be the ample opportunity for self-referential information to manifest, thereby amplifying the self-reference processing. Additionally, both designs appear to reinforce the association between self-referential information and the task-irrelevant dimension. This finding suggests that the reduction in the CE stems precisely from the presence of self-referential information.

During the time interval of 200 to 250 ms, a distinct left central N2 amplitude can be observed. The N2 amplitude during self-referential conditions is smaller than that during non-self-referential conditions. The N2 is believed to signify early higher-order operations related to stimulus discrimination and categorization^18^. Interestingly, some authors have established the N2 as the temporal boundary between automatic and controlled processing phases^35^. N2 is related to attentional resources and reflects their input^36,37^. Therefore, elaborative processing of self-relevance would be anticipated to occur during subsequent cognitive processing stages^38^. Previous studies have found that the importance of self-referential information can lead to smaller N2 amplitude^14,16,17^. These findings indicate that self-relevant content is more easily retrievable, and top-down cognitive resources are less consumed. In the present study, the N2 may indicate the preliminary identification of self-referential information, consume fewer cognitive resources, and engage in elaborative processing when compared to non-self-referential conditions.

Interestingly, the MVPA results reveal reliable decoding of self-referential information in the early time window after stimulus presentation, corresponding to N2 amplitude. Comparing ERP and MVPA results of self-reference processing, we observe that the time range of above-chance decoding extends significantly beyond the N2 time window. This suggests that self-referential processing, besides involving early recognition, may also encompass conflict monitoring. In our study, both ERP and MVPA results consistently reveal a leftward bias in the self-reference effect. This finding aligns with prior research on collective self-referential processing^38,39^. They found that the self-reference effect was more obvious on the left electrode sites when using stimuli (such as national flags and forenames) to represent collective self-reference. Additionally, an fMRI study revealed left lateralization in the processing of the relational and collective self^40^. However, it’s essential to acknowledge that numerous studies emphasize the crucial role of the right hemisphere in self-reference processing^41–43^. These investigations primarily focus on the self-concept associated with individual self. In our study, stimulus-induced (my) self-referential processing may lean more toward collective-self reference. However, self-reference processing is a complex neural phenomenon, drawing from diverse sources^44,45^. The leftward bias doesn’t imply that self-referential information arises solely from a fixed neural source.

N450 is considered to reflect conflict monitoring^20,46^. Our findings indicate that the N450 amplitude is more pronounced in incongruent conditions compared to congruent ones, regardless of self-referential information. However, it would be premature to conclude that self-reference information does not impact the conflict monitoring process. Larson et al.^20^ propose that the N450 may reflect underlying neural processes that are consistent (i.e., more automatic) regardless of the amount of top-down control needed. Additionally, Yin et al.^4^ suggest that self-referential information indeed influences goal-based top-down attention. Thus, while self-reference information likely plays a role in conflict monitoring, its effects may not be directly observable in the N450 amplitude. Notably, MVPA is more sensitive than ERP analysis and can depict the changes of neural activity with whole-brain features^26,29,47,48^. According to MVPA results, self-referential information can be decoded in the early stages, yet during the mid-phase of the response, precisely at the crossroads of self-referential and congruency effects, there is a decline in the decoding ability of self-referential information. Meanwhile, the decoding ability of congruency information rises, indicating that self-referential processing concludes relatively earlier compared to the persistence of the congruency effect until the end of the response. Therefore, the self-referential effect is not entirely distinct but rather exhibits some overlap with the congruency effect. Interestingly, the decoding of congruency reveals that self-reference conditions can decode conflicts earlier than non-self-reference conditions. This discrepancy may arise from ERP technology’s limitations in capturing nuanced influences, whereas MVPA provides a more sensitive lens for examining self-reference effects on conflict monitoring.

SP is considered to be conflict resolution^49–51^ and also reflect attention allocation^20,23,50^. Specifically, Coderre et al.^49^ found that at a −400 ms stimulus onset asynchrony (SOA), SP patterns occur in Stroop and inhibition effects but not during facilitation effects. This suggests that the SP amplitude is related to conflict resolution. Meanwhile, Larson et al.^20^ found that the parietal conflict SP amplitude monotonically decreased with lesser degrees of conflict. In simpler terms, the greater the conflict, the larger the SP. Our study suggests that the SP amplitude is smaller under the self-referential condition. This may indicate that the conflict level in the self-referential condition is lower, requiring fewer attentional resources for resolution. Surprisingly, reliable decoding of self-referential information is not observed in the conflict resolution, which apparently contradicts the SP results. Reliable decoding of congruency is observed in conflict resolution. This discrepancy may be because the later stage has reached the conflict resolution stage, in which case top-down processing comes into play, and self-referential processing occurs in the early stage.

Self-referential information plays a significant role in conflict control processes. Two aspects of the processing of self-referential information in the Stroop task deserve further consideration. On the one hand, in the Stroop task, incongruent trials present a word meaning that differs from the ink color. The task-irrelevant dimension (word meaning) and task-relevant dimension (ink color) activate in parallel in different processing channels^52,53^. Due to higher connection weight, word meaning has a processing advantage over the ink color. When self-referential information is presented, it tends to be associated with a stronger activation dimension (word meaning). On the other hand, self-referential information and task-irrelevant dimension involve semantic processing, while the task-relevant dimension pertains to visual processing^54^. Self-referential information activates semantic representations of self-concept, such as personal characteristics, preferences, or memories^55,56^. When self-referential information is identified, it may form a coherent semantic unit with task-irrelevant dimension (word meaning), which is more easily bound than task-relevant dimensions (ink color). Considering these two factors, self-referential information should be bound to task-irrelevant dimensions.

Moreover, self-referential information can be easily distinguished and retrieved in the early stages of cognitive processing, thereby conserving top-down cognitive resources^14,16,17^. This benefit means more cognitive resources are available to actively inhibit task-irrelevant information, compared to non-self-referential situations. In fact, the involvement of self-referential information in the early stages of processing may lead to the earlier emergence of task-irrelevant dimension. This early activation provides an opportunity for active inhibition of task-irrelevant information^9,11,57^. If the task-irrelevant dimension presented in advance is efficiently encoded and inhibited, processing of the goal response is promoted because the distractor-related representation is actively inhibited^58^. This active inhibition of task-irrelevant information contributes to a decrease in the congruency effect, ultimately resulting in more effective conflict resolution. Specifically, prior activation of inhibitory control helps to suppress the output of irrelevant response representation, ultimately decreasing conflict by reducing interference with the target response process^9^.

The PDP model further posits that the task-irrelevant dimension (word meaning) and task-relevant dimension (ink color) are activated in parallel in different processing channels^52^. Early processing of the task-irrelevant dimension may lessen the overlap and competition between task-relevant and task-irrelevant dimensions, making it easier to filter out irrelevant information when striving to achieve the goal. When the activation of the distractor dimension is suppressed in advance, the task-relevant dimension does not require excessive strength to compete, allowing for quicker conflict monitoring and lessened conflict intensity. During the conflict resolution phase, the early processing of self-referential information has already reduced the competition between task-irrelevant and task-relevant dimensions. Consequently, individuals experience less interference when facing conflicts, leading to a decrease in conflict intensity (as reflected by a smaller SP)^20^. Since early processing of self-referential information weakens the competition between task-irrelevant and task-relevant dimensions, there is less need for additional cognitive resources to suppress or exclude irrelevant information during conflict resolution. This, in turn, reduces the cognitive resources required for conflict resolution, leading to a more efficient cognitive system overall. Furthermore, the early processing of self-referential information may lead to a more efficient cognitive system in subsequent stages, actively inhibiting irrelevant dimensions, and achieving higher efficiency with lower resource consumption during conflict resolution. In summary, self-referential information significantly influences conflict control processes by inhibiting the activation of task-irrelevant dimensions and reducing conflict intensity through efficient cognitive resource allocation.

Given the previously discussed role of self-referential information in conflict processing, we now revisit its impact within the distinct frameworks of block and event-based designs. In block designs, it reduces the CE. This reduction is likely attributable to the fact that participants are not required to process the self-referential information on every trial, thereby affording them sufficient time to allocate attentional resources towards conflict resolution. However, in event-based designs, self-referential information does not reduce the CE. This could be due to the necessity for participants to process such information in every trial, which may not leave sufficient time for the mobilization of attentional resources. In general, self-referential information takes time to exert its influence in Stroop tasks, thus facilitating conflict resolution. Additionally, Li et al.^12^ in their Experiment 1 (event-base design) demonstrated that self-referential information from a preceding trial can reduce the CE in the current trial, suggesting that self-referential information may require a latency period to achieve its maximal impact.

In the present research, we employ a single Chinese character (wo de) to represent the concept of self. Although Chinese self-relevant possessive pronouns can active self-referential processing^59^, it is essential to incorporate a variety of stimuli to avoid confounding effects related to the physical properties of the stimulus. These multiple materials about self-relevant information include names^38^, faces^60,61^, and handwriting^13^. Moreover, integrating the third person into the study could offer valuable insights into non-self-reference situations, utilizing terms like “his” and “her.” Such an approach ensures a more comprehensive examination of self-referential information and avoids the confounding of the stimulus property. Regarding the neural regions involved in self-referential processing, our findings suggest a leftward bias, consistent with previous research on the collective self^38,39^. However, it’s important to acknowledge that EEG has inherent limitations in precisely localizing brain activity. Therefore, future investigations could incorporate functional magnetic resonance imaging (fMRI) to precisely localize the brain regions involved during these tasks.

This study provides the first neurophysiological evidence for the role of self-referential information in conflict processing. By combining ERP with MVPA, we identified self-referential information, indexed by above-chance decoding accuracy and comparable N2 between self-reference and non-self-reference conditions. Notably, Self-referential information detected conflicts earlier than non-self-referential information, indexed by above-chance decoding. During the conflict resolution stage, cognitive control recruited fewer cognitive resources to resolve conflicts, indexed by comparable SP between self-reference conditions and non-self-reference conditions in incongruent situations.

## Methods

### Subjects

We conducted a priori power analysis using G*Power 3.1^62^. With a desired power of 0.80, a significance level of 0.05, and an effect size of f = 0.25, the power analysis suggested an estimated sample size of approximately 24 participants. Thirty-three healthy participants, including 20 females and 13 males with a mean age of 20.84 ± 2.18 years (range: 18–29 years) from the local city participated in this experiment. All participants were right-handed and had normal or corrected-to-normal vision. All participants completed a questionnaire regarding their gender identity, with a free-response option to indicate male, female, or other. This study received approval from the Human Ethics Committee of Southwest University in China (ID: H20069). The ethical principles outlined in the Declaration of Helsinki were strictly adhered to throughout the study. Measures were taken to protect the privacy and confidentiality of all participants’ data. Prior to the experiment, written informed consent was obtained from all participants

### Procedure and task design

The experimental procedure was conducted on a 19-inch monitor (1280 ×1024 pixels, 60 Hz refresh rate), with stimulus presentation and data acquisition performed using E-Prime software 2.0.10. The modified Color-Stroop task was used in the experiment, where stimuli consisted of four Chinese color words and the carrier words for blue, red, green, and yellow (represented by the characters “lan,” “hong,” “lv,” and “huang” in Chinese), the possessive pronoun “my” (Chinese for “wo de”), and the definite article “the” (Chinese for “zhe ge”). All stimuli were centrally presented on the screen using a 35-point Courier New font.

**Fig. 4:**
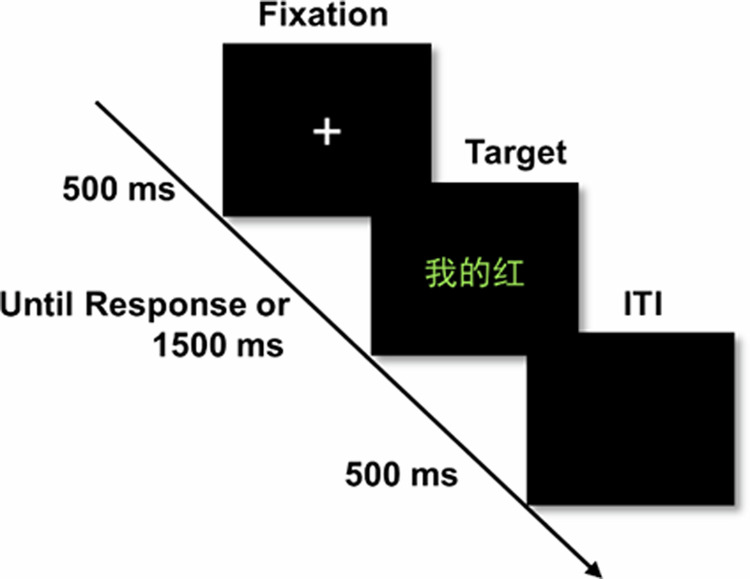
Task design. The sequence of a typical in the modified Stroop task. The Chinese character means the English character “my red”.

As depicted in Fig. 4, each trial was initiated with the presentation of a 500 ms fixation cross, followed by the presentation of stimuli (i.e., pronoun and color word). The stimulus was terminated with the key press. The participants had to respond to the ink color of the word as quickly and accurately as possible while disregarding the word’s meaning before a 1500 ms response deadline. In addition, the stimulus presentation lasted for 1500 ms in the absence of a participant key press. Consecutive trials were separated by a 500 ms interval. They were subsequently instructed to press “D” with the left index finger, “F” with the left middle finger, “J” with the right index finger, or “K” with the right middle finger. 15 participants were assigned such that the ink colors “red,” “green,” “yellow,” and “blue” respectively correspond to the “D,” “F,” “J,” and “K” keys on the keyboard. For the remaining 18 participants, the ink colors “red,” “green,” “yellow,” and “blue” respectively correspond to the “K,” “J,” “F,” and “D” keys on the keyboard.

### Experimental design and statistical analyses

The experiment employed a 2 (congruency: congruent vs. incongruent) × 2 (self-referential information: the self-reference condition “my” vs. the non-self-reference condition “the”) design. Prior to the formal experiment, participants completed a practice block consisting of 32 trials. Only when the average accuracy rate in the practice block exceeded 90% did the formal experiment commence. The experiment comprised a total of 582 trials (6 blocks, 97 trials per block). Considering that the first trial of each block was excluded from further analysis, the total number of trials used for analysis amounted to 576 (6 blocks, 96 trials per block). The study employed a blocked design, with 3 blocks of the self-reference condition and 3 blocks of the non-self-reference condition. The block of self-reference condition contained two trial types (i.e., congruent with self-reference and incongruent with self-reference) which were presented equally 48 times. The block of the non-self-reference condition contained two trial types (i.e., congruent with non-self-reference and incongruent with non-self-reference) which were presented equally 48 times. All trials within each block were pseudo-randomly generated. To ensure a balanced order, the ABBA procedure was implemented for the sequence of the 6 blocks. Specifically, the ABBA sequence (from the first block to the sixth block) meant: self/non-self/non-self/self/self/non-self, or non-self/self/self/non-self/non-self/self. This ensured a systematic alternation between self and non-self-conditions in the sequence of blocks.

During the behavioral analysis, we excluded the first trial of each block and any trials with errors or post-error responses. Additionally, trials with RTs exceeding 3 SDs above or below the mean RT were excluded from further analysis. The RT and accuracy were analyzed using repeated-measures ANOVA, with within-subject factors of congruency (congruent vs. incongruent) and self-referential information (the self-reference condition “my” vs. the non-self-reference condition “the”), respectively. The unit for the RT was milliseconds.

### EEG recording and preprocessing

EEG data were collected using standard 64 in-cap Ag/AgCl electrodes following the extended international 10–20 system (Brain Products). Two additional electrodes were placed over the left and right mastoids. Vertical and horizontal electrooculograms (VEOG and HEOG) were recorded from below the right eye and over the outer canthus of the left eye. During data acquisition, all electrodes were referenced to the electrode FCz, while the ground electrode was positioned at AFz. EEG data were continuously collected at a sampling rate of 500 Hz and online filtered at 0.1–100 Hz. The impedance of all electrodes remained at 5 kΩ throughout the recording.

After data acquisition, EEG data preprocessing was performed using EEGLAB 19.0^63^ and MATLAB R2017b. Offline data were referenced to the mean of the left and right mastoids (TP9, TP10). Data were filtered with 0.1–30 Hz bandpass filter using a basic finite impulse response filter. Continuous EEG data were segmented from −100 to 1000 ms using epochs locked on the stimulus markers. The segmented data were baseline-corrected using a −100 to 0 ms baseline window. After extracting the epochs, we removed error trials and post-error trials from subsequent analyses to separate the cognitive processes involved in resolving conflict from those related to error processing. For the removal of electro-oculogram artifacts, we used an independent component analysis (ICA) approach to correct eye movements and blinks^64^. Automated rejection of epochs was performed whenever the voltage exceeded 100 μV.

### ERP analysis

Based on previous studies and grand mean mapping, the stimuli-locked N2 amplitude was calculated at left central electrodes (C3, C5, CP3, CP5) using time windows of 50 ms (200 to 250 ms). The stimuli-locked N450 amplitude were calculated at centro-parietal electrodes (CPz, P1, P2, Pz) using time windows of 130 ms (350 to 480 ms). Additionally, the stimuli-locked SP amplitude was calculated at centro-parietal electrodes (CP1, CP2, CPz) using time windows of 150 ms (600 to 750 ms). These event-related potential (ERP) amplitudes were analyzed using repeated-measures ANOVA, with within-subject factors of congruency (congruent, incongruent) and self-referential information (self-reference and non-self-reference condition).

### MVPA

Due to the higher sensitivity of multivariate analyses in decoding higher-order cognitive processes, we re-preprocessed the stimulus-locked data to apply MVPA. Offline data were referenced to the average of both mastoids and filtered at 0.1–30 Hz. Continuous data were segmented from −100 to 1000 ms and baseline-corrected for each epoch. Error trials and post-error trials were excluded after epoch extraction. Research suggests that classifiers can learn to ignore bad channels or suppress noise during training, potentially making artifact correction unnecessary in decoding analysis^26^. Thus, no artifact correction was performed during the re-preprocessing of the EEG data. The experimental trials were classified into four trial types based on congruency (i.e., congruent and incongruent) and self-referential information (i.e., self-reference and non-self-reference conditions). Multivariate Pattern Analysis (MVPA) leveraged a variety of machine learning classifiers for discrimination, including Support Vector Machines (SVM), Gaussian Naive Bayes (GNB), and Linear Discriminant Analysis (LDA)^26^. In this study, we used a backward decoding classification algorithm (linear discriminant analysis, LDA), with two of the four trial types as classes (i.e., self-reference-incongruent and self-reference-congruent, non-self-reference-congruent and non-self-reference-incongruent, congruent-self-reference and congruent-non-self-reference, incongruent-self-reference and incongruent-non-self-reference), and 60 electrodes (excluding HEOG, VEOG, and two reference electrodes) were used as features. The analysis aimed to test whether the classifier could discriminate between different types of self-referential and congruency conditions based on the divergent EEG patterns following each single-trial response.

To achieve these four discriminations, the Amsterdam Decoding and Modeling toolbox (ADAM) was used for the re-preprocessed EEG data^32^. We resampled the data at 50 Hz to reduce the time consumption of the decoding algorithms. A leave-one-out procedure was used. Specifically, the classifier was trained on four folds and tested on the remaining fold. This procedure was repeated 5 times until all the data chunks had been tested. To minimize any potential biases resulting from the assignment of trials to different groups, we performed 10 iterations of the entire procedure, shuffling the order of the trials at the beginning of each iteration^65^. We used The Area Ander the ROC Curve (AUC) as a reliable measure of classification accuracy, where higher AUC values indicated better classification performance^32^. Topographical maps were generated using classifier weights assigned to individual electrode channels. To explore the topographical distribution of neural activity associated with significant classification, we computed these maps by multiplying the classifier weights of all electrodes with the covariance matrix of the data across electrodes^32,33,66^. For unbalanced trial numbers within a class, the majority class was under-sampled to avoid classification bias^32^.

### Reporting summary

Further information on research design is available in the Nature Research Reporting Summary linked to this article.

### Supplementary information

Reporting Summary

## Data Availability

The data will be made available upon request to the corresponding author with a formal data-sharing agreement. There are no limits on data sharing.

## Code availability

The software and code used in the study are open-source and publicly available from the corresponding authors upon request for academic purposes.

## References

[1] Miller, E. K. & Cohen, J. D. An integrative theory of prefrontal cortex function. *Annu. Rev. Neurosci.* **24**, 167–202 (2001). 11283309 10.1146/annurev.neuro.24.1.167

[2] Stroop, J. R. Studies of interference in serial verbal reactions. *J. Exp. Psychol.* **18**, 643–662 (1935). 10.1037/h0054651

[3] Dignath, D., Eder, A. B., Herbert, C. & Kiesel, A. Self-related primes reduce congruency effects in the Stroop task. *J. Exp. Psychol. Gen* **151**, 2879–2892 (2022). 10.1037/xge0001210 35604709

[4] Yin, S., Sui, J., Chiu, Y.-C., Chen, A. & Egner, T. Automatic prioritization of self-referential stimuli in working memory. *Psychol. Sci.* **30**, 415–423 (2019). 30653399 10.1177/0956797618818483

[5] Yin, S., Bi, T., Chen, A. & Egner, T. Ventromedial prefrontal cortex drives the prioritization of self-associated stimuli in working memory. *J. Neurosci.* **41**, 2012–2023 (2021). 33462089 10.1523/JNEUROSCI.1783-20.2020 PMC7939096

[6] Alexopoulos, T., Muller, D., Ric, F. & Marendaz, C. I. me, mine: Automatic attentional capture by self-related stimuli: Attention to self-related stimuli. *Eur. J. Soc. Psychol.* **42**, 770–779 (2012). 10.1002/ejsp.1882

[7] Sui, J., He, X., Golubickis, M., Svensson, S. L. & Neil Macrae, C. Electrophysiological correlates of self-prioritization. *Conscious. Cogn.* **108**, 103475 (2023). 36709725 10.1016/j.concog.2023.103475

[8] Banaji, M. R. & Prentice, D. A. The self in social contexts. *Annu. Rev. Psychol.* **45**, 297–332 (1994). 10.1146/annurev.ps.45.020194.001501

[9] Chao, H.-F. Active inhibition of a distractor word: The distractor precue benefit in the Stroop color-naming task. *J. Exp. Psychol.: Hum. Percept. Perform.* **37**, 799–812 (2011). 21480743 10.1037/a0022191

[10] Dyer, F. N. The duration of word meaning responses: Stroop interference for different preexposures of the word. *Psychon. Sci.* **25**, 229–231 (1971). 10.3758/BF03329102

[11] Glaser, M. O. & Glaser, W. R. Time course analysis of the Stroop phenomenon. *J. Exp. Psychol.: Hum. Percept. Perform.* **8**, 875–894 (1982). 6218237 10.1037//0096-1523.8.6.875

[12] Li, Z., Chen, Y., Yin, S. & Chen, A. Self-referential information optimizes conflict adaptation. *Mem. Cogn* **52**, 648–662 (2024). 10.3758/s13421-023-01490-8 38261248

[13] Chen, A. et al. The temporal features of self-referential processing evoked by Chinese handwriting. *J. Cogn. Neurosci.* **20**, 816–827 (2008). 18201135 10.1162/jocn.2008.20505

[14] Chen, J. et al. Temporal features of the degree effect in self-relevance: Neural correlates. *Biol. Psychol.* **87**, 290–295 (2011). 21470572 10.1016/j.biopsycho.2011.03.012

[15] Guan, L., Qi, M., Zhang, Q. & Yang, J. The neural basis of self-face recognition after self-concept threat and comparison with important others. *Soc. Neurosci.* **9**, 424–435 (2014). 24852316 10.1080/17470919.2014.920417

[16] Muñoz, F. et al. Neural dynamics in the processing of personal objects as an Index of the brain representation of the self. *Brain Topogr.* **33**, 86–100 (2020). 31776831 10.1007/s10548-019-00748-2

[17] Xu, K. et al. Importance modulates the temporal features of self-referential processing: An event-related potential study. *Front. Hum. Neurosci*. **11**, 470 (2017). 10.3389/fnhum.2017.00470 PMC5613165 28983245

[18] Patel, S. H. & Azzam, P. N. Characterization of N200 and P300: Selected studies of the event-related potential. *Int J. Med Sci.* **2**, 147–154 (2005). 16239953 10.7150/ijms.2.147 PMC1252727

[19] Kousaie, S. & Phillips, N. A. Conflict monitoring and resolution: Are two languages better than one? Evidence from reaction time and event-related brain potentials. *Brain Res.* **1446**, 71–90 (2012). 22356886 10.1016/j.brainres.2012.01.052

[20] Larson, M. J., Kaufman, D. A. S. & Perlstein, W. M. Neural time course of conflict adaptation effects on the Stroop task. *Neuropsychologia* **47**, 663–670 (2009). 19071142 10.1016/j.neuropsychologia.2008.11.013

[21] West, R. & Alain, C. Effects of task context and fluctuations of attention on neural activity supporting performance of the Stroop task. *Brain Res.* **873**, 102–111 (2000). 10915815 10.1016/S0006-8993(00)02530-0

[22] Chen, A., Bailey, K., Tiernan, B. N. & West, R. Neural correlates of stimulus and response interference in a 2–1 mapping stroop task. *Int. J. Psychophysiol.* **80**, 129–138 (2011). 21356252 10.1016/j.ijpsycho.2011.02.012

[23] Larson, M. J., Clayson, P. E. & Clawson, A. Making sense of all the conflict: A theoretical review and critique of conflict-related ERPs. *Int. J. Psychophysiol.* **93**, 283–297 (2014). 24950132 10.1016/j.ijpsycho.2014.06.007

[24] Tang, D., Hu, L., Li, H., Zhang, Q. & Chen, A. The neural dynamics of conflict adaptation within a look-to-do transition. *PLOS ONE* **8**, e57912 (2013). 23469102 10.1371/journal.pone.0057912 PMC3585284

[25] West, R., Jakubek, K., Wymbs, N., Perry, M. & Moore, K. Neural correlates of conflict processing. *Exp. Brain Res* **167**, 38–48 (2005). 16082533 10.1007/s00221-005-2366-y

[26] Grootswagers, T., Wardle, S. G. & Carlson, T. A. Decoding dynamic brain patterns from evoked responses: A tutorial on multivariate pattern analysis applied to time series neuroimaging data. *J. Cogn. Neurosci.* **29**, 677–697 (2017). 27779910 10.1162/jocn_a_01068

[27] Kaiser, D., Oosterhof, N. N. & Peelen, M. V. The neural dynamics of attentional selection in natural scenes. *J. Neurosci.* **36**, 10522–10528 (2016). 27733605 10.1523/JNEUROSCI.1385-16.2016 PMC6601932

[28] Li, Q. et al. Not all errors are created equal: decoding the error-processing mechanisms using alpha oscillations. *Cereb. Cortex* **33**, 8110–8121 (2023). 36997156 10.1093/cercor/bhad102

[29] Bae, G.-Y. & Luck, S. J. Dissociable decoding of spatial attention and working memory from EEG oscillations and sustained potentials. *J. Neurosci.* **38**, 409–422 (2018). 29167407 10.1523/JNEUROSCI.2860-17.2017 PMC5761617

[30] Bae, G.-Y. & Luck, S. J. Reactivation of previous experiences in a working memory task. *Psychol. Sci.* **30**, 587–595 (2019). 30817224 10.1177/0956797619830398 PMC6472175

[31] Fahrenfort, J. J., van Leeuwen, J., Olivers, C. N. L. & Hogendoorn, H. Perceptual integration without conscious access. *Proc. Natl Acad. Sci.* **114**, 3744–3749 (2017). 28325878 10.1073/pnas.1617268114 PMC5389292

[32] Fahrenfort, J. J., van Driel, J., van Gaal, S. & Olivers, C. N. L. From ERPs to MVPA using the Amsterdam Decoding and Modeling Toolbox (ADAM). *Front. Neurosci.* **12**, 368 (2018). 30018529 10.3389/fnins.2018.00368 PMC6038716

[33] Haufe, S. et al. On the interpretation of weight vectors of linear models in multivariate neuroimaging. *NeuroImage* **87**, 96–110 (2014). 24239590 10.1016/j.neuroimage.2013.10.067

[34] Fan, W. et al. Negative Emotion weakens the degree of self-reference effect: Evidence from ERPs. *Front. Psychol*. **7**, 1408 (2016). 10.3389/fpsyg.2016.01408 PMC5039173 27733836

[35] Carretié, L., Hinojosa, J. A., Martín-Loeches, M., Mercado, F. & Tapia, M. Automatic attention to emotional stimuli: Neural correlates. *Hum. Brain Mapp.* **22**, 290–299 (2004). 15202107 10.1002/hbm.20037 PMC6871850

[36] Daffner, K. R. et al. Regulation of attention to novel stimuli by frontal lobes: an event-related potential study. *NeuroReport* **9**, 787 (1998). 9579666 10.1097/00001756-199803300-00004

[37] Folstein, J. R. & Van Petten, C. Influence of cognitive control and mismatch on the N2 component of the ERP: A review. *Psychophysiology* **45**, 152–170 (2007). 10.1111/j.1469-8986.2007.00602.x PMC2365910 17850238

[38] Chen, J., Zhang, Y., Zhong, J., Hu, L. & Li, H. The primacy of the individual versus the collective self: Evidence from an event-related potential study. *Neurosci. Lett.* **535**, 30–34 (2013). 23313591 10.1016/j.neulet.2012.11.061

[39] Fan, W. et al. The temporal features of self-referential processing evoked by national flag. *Neurosci. Lett.* **505**, 233–237 (2011). 22015762 10.1016/j.neulet.2011.10.017

[40] Zheng, Y., Xiao, Z., Wei, L. & Chen, H. The Neural Representation of Relational- and Collective-Self: Two Forms of Collectivism. *Front. Psychol*. **9**, 2624 (2018). 10.3389/fpsyg.2018.02624 PMC6312116 30619016

[41] Han, S. & Northoff, G. Culture-sensitive neural substrates of human cognition: a transcultural neuroimaging approach. *Nat. Rev. Neurosci.* **9**, 646–654 (2008). 18641669 10.1038/nrn2456

[42] Han, S. & Northoff, G. Understanding the self: a cultural neuroscience approach. in *Progress in Brain Research* (ed. Chiao, J. Y.) vol. 178 203–212 (Elsevier, 2009). 10.1016/S0079-6123(09)17814-7 19874971

[43] Keenan, J. P., Wheeler, M. A., Gallup, G. G. & Pascual-Leone, A. Self-recognition and the right prefrontal cortex. *Trends Cogn. Sci.* **4**, 338–344 (2000). 10962615 10.1016/S1364-6613(00)01521-7

[44] Northoff, G. et al. Self-referential processing in our brain—A meta-analysis of imaging studies on the self. *NeuroImage* **31**, 440–457 (2006). 16466680 10.1016/j.neuroimage.2005.12.002

[45] Northoff, G. & Bermpohl, F. Cortical midline structures and the self. *Trends Cogn. Sci.* **8**, 102–107 (2004). 15301749 10.1016/j.tics.2004.01.004

[46] Heidlmayr, K., Kihlstedt, M. & Isel, F. A review on the electroencephalography markers of Stroop executive control processes. *Brain Cogn.* **146**, 105637 (2020). 33217721 10.1016/j.bandc.2020.105637

[47] Stokes, M. G., Wolff, M. J. & Spaak, E. Decoding rich spatial information with high temporal resolution. *Trends Cogn. Sci.* **19**, 636–638 (2015). 26440122 10.1016/j.tics.2015.08.016 PMC4636428

[48] van Ede, F., Chekroud, S. R., Stokes, M. G. & Nobre, A. C. Decoding the influence of anticipatory states on visual perception in the presence of temporal distractors. *Nat. Commun.* **9**, 1449 (2018). 29654312 10.1038/s41467-018-03960-z PMC5899132

[49] Coderre, E., Conklin, K. & van Heuven, W. J. B. Electrophysiological measures of conflict detection and resolution in the Stroop task. *Brain Res.* **1413**, 51–59 (2011). 21840503 10.1016/j.brainres.2011.07.017

[50] Donohue, S. E., Appelbaum, L. G., McKay, C. C. & Woldorff, M. G. The neural dynamics of stimulus and response conflict processing as a function of response complexity and task demands. *Neuropsychologia* **84**, 14–28 (2016). 26827917 10.1016/j.neuropsychologia.2016.01.035 PMC4808442

[51] Heidlmayr, K., Hemforth, B., Moutier, S. & Isel, F. Neurodynamics of executive control processes in bilinguals: evidence from ERP and source reconstruction analyses. *Front. Psychol*. **6**, 821 (2015). 10.3389/fpsyg.2015.00821 PMC4467069 26124740

[52] Cohen, J. D., Dunbar, K. & McClelland, J. L. On the control of automatic processes: A parallel distributed processing account of the Stroop effect. *Psychol. Rev.* **97**, 332–361 (1990). 2200075 10.1037/0033-295X.97.3.332

[53] MacLeod, C. M. Half a century of research on the Stroop effect: An integrative review. *Psychol. Bull.* **109**, 163–203 (1991). 2034749 10.1037/0033-2909.109.2.163

[54] Parris, B. A., Hasshim, N., Wadsley, M., Augustinova, M. & Ferrand, L. The loci of Stroop effects: a critical review of methods and evidence for levels of processing contributing to color-word Stroop effects and the implications for the loci of attentional selection. *Psychol. Res.* **86**, 1029–1053 (2022). 34389901 10.1007/s00426-021-01554-x PMC9090875

[55] Sui, J. Self-reference acts as a golden thread in binding. *Trends Cogn. Sci.* **20**, 482–483 (2016). 27315761 10.1016/j.tics.2016.04.005 PMC6029663

[56] Sui, J. & Humphreys, G. W. The integrative self: How self-reference integrates perception and memory. *Trends Cogn. Sci.* **19**, 719–728 (2015). 26447060 10.1016/j.tics.2015.08.015

[57] Roelofs, A. Attention, temporal predictability, and the time course of context effects in naming performance. *Acta Psychol.* **133**, 146–153 (2010). 10.1016/j.actpsy.2009.11.003 19963201

[58] Chen, Q., Meng, Z., Xu, L., Hou, Y. & Chen, A. Effective connectivity analysis reveals the time course of the Stroop effect in manual responding. *Biol. Psychol.* **178**, 108526 (2023). 36841469 10.1016/j.biopsycho.2023.108526

[59] Zhou, A. et al. An ERP study on the effect of self-relevant possessive pronoun. *Neurosci. Lett.* **480**, 162–166 (2010). 20561562 10.1016/j.neulet.2010.06.033

[60] Sui, J., Hong, Y., Hong Liu, C., Humphreys, G. W. & Han, S. Dynamic cultural modulation of neural responses to one’s own and friend’s faces. *Soc. Cogn. Affect. Neurosci.* **8**, 326–332 (2013). 22258798 10.1093/scan/nss001 PMC3594724

[61] Sui, J., Zhu, Y. & Han, S. Self-face recognition in attended and unattended conditions: An event-related brain potential study. *NeuroReport* **17**, 423 (2006). 16514370 10.1097/01.wnr.0000203357.65190.61

[62] Faul, F., Erdfelder, E., Lang, A.-G. & Buchner, A. G*Power 3: A flexible statistical power analysis program for the social, behavioral, and biomedical sciences. *Behav. Res. Methods* **39**, 175–191 (2007). 17695343 10.3758/BF03193146

[63] Delorme, A. & Makeig, S. EEGLAB: an open source toolbox for analysis of single-trial EEG dynamics including independent component analysis. *J. Neurosci. Methods* **134**, 9–21 (2004). 15102499 10.1016/j.jneumeth.2003.10.009

[64] Jutten, C. & Herault, J. Blind separation of sources, part I: An adaptive algorithm based on neuromimetic architecture. *Signal Process.* **24**, 1–10 (1991). 10.1016/0165-1684(91)90079-X

[65] de Vries, I. E. J., van Driel, J. & Olivers, C. N. L. Decoding the status of working memory representations in preparation of visual selection. *NeuroImage* **191**, 549–559 (2019). 30840904 10.1016/j.neuroimage.2019.02.069

[66] Takacs, A., Mückschel, M., Roessner, V. & Beste, C. Decoding stimulus–response representations and their stability using EEG-based multivariate pattern analysis. *Cereb. Cortex Commun.* **1**, tgaa016 (2020). 34296094 10.1093/texcom/tgaa016 PMC8152870

