# Supplementary material for: Attenuated conflict self-referential information facilitating conflict resolution
Source: NPJ Sci Learn. 2024 Jul 19;9:47. doi: 10.1038/s41539-024-00256-4 (PMC11271533; doi:10.1038/s41539-024-00256-4)
Supplement: Supplementary file 1 — Reporting Summary [file 41539_2024_256_MOESM1_ESM.pdf]

nature portfolio

Corresponding author(s): DAPR, NPJSCILEARN-00893R1

Last updated by author(s): May 9, 2024

Reporting Summary

Nature Portfolio wishes to improve the reproducibility of the work that we publish. This form provides structure for consistency and transparency in reporting. For further information on Nature Portfolio policies, see our Editorial Policies and the Editorial Policy Checklist.

Statistics

For all statistical analyses, confirm that the following items are present in the figure legend, table legend, main text, or Methods section.

n/a	Confirmed	
☐	☒	The exact sample size (*n*) for each experimental group/condition, given as a discrete number and unit of measurement
☐	☒	A statement on whether measurements were taken from distinct samples or whether the same sample was measured repeatedly
☐	☒	The statistical test(s) used AND whether they are one- or two-sided<br>*Only common tests should be described solely by name; describe more complex techniques in the Methods section.*
☐	☒	A description of all covariates tested
☐	☒	A description of any assumptions or corrections, such as tests of normality and adjustment for multiple comparisons
☐	☒	A full description of the statistical parameters including central tendency (e.g. means) or other basic estimates (e.g. regression coefficient) AND variation (e.g. standard deviation) or associated estimates of uncertainty (e.g. confidence intervals)
☐	☒	For null hypothesis testing, the test statistic (e.g. *F*, *t*, *r*) with confidence intervals, effect sizes, degrees of freedom and *P* value noted<br>*Give P values as exact values whenever suitable.*
☒	☐	For Bayesian analysis, information on the choice of priors and Markov chain Monte Carlo settings
☒	☐	For hierarchical and complex designs, identification of the appropriate level for tests and full reporting of outcomes
☐	☒	Estimates of effect sizes (e.g. Cohen's *d*, Pearson's *r*), indicating how they were calculated

Our web collection on statistics for biologists contains articles on many of the points above.

Software and code

Policy information about availability of computer code

Data collection	The data will be made available upon request to the corresponding author with a formal data sharing agreement. EEGLAB19.0, MATLAB R2017b,ADAM
Data analysis	The software and code used in the study are open-source and publicly available.

For manuscripts utilizing custom algorithms or software that are central to the research but not yet described in published literature, software must be made available to editors and reviewers. We strongly encourage code deposition in a community repository (e.g. GitHub). See the Nature Portfolio guidelines for submitting code & software for further information.

Data

Policy information about availability of data

All manuscripts must include a data availability statement. This statement should provide the following information, where applicable:

- Accession codes, unique identifiers, or web links for publicly available datasets
- A description of any restrictions on data availability
- For clinical datasets or third party data, please ensure that the statement adheres to our policy

The data will be made available upon request to the corresponding author with a formal data sharing agreement. The software and code used in the study are open-source and publicly available.

Research involving human participants, their data, or biological material

Policy information about studies with human participants or human data. See also policy information about sex, gender (identity/presentation), and sexual orientation and race, ethnicity and racism.

Reporting on sex and gender	All participants completed a questionnaire regarding their gender identity, with a free-response option to indicate male, female, or other.
Reporting on race, ethnicity, or other socially relevant groupings	No reporting on race, ethnicity,or other social relevant
Population characteristics	We describe the age of population
Recruitment	Recruitment through online groups (e.g. QQ in China)
Ethics oversight	This study received approval from the Human Ethics Committee of local University in China[illegible]H20069)

Note that full information on the approval of the study protocol must also be provided in the manuscript.

Field-specific reporting

Please select the one below that is the best fit for your research. If you are not sure, read the appropriate sections before making your selection.

☐ Life sciences ☒ Behavioural & social sciences ☐ Ecological, evolutionary & environmental sciences

For a reference copy of the document with all sections, see nature.com/documents/nr-reporting-summary-flat.pdf

Behavioural & social sciences study design

All studies must disclose on these points even when the disclosure is negative.

Study description	Dignath et al. (2022) found that self-referential primes reduced the CE. They proposed that self-referential information can act as a signal to enhance cognitive control, but cannot deny the possibility that self-referential information may attract and hold attention. However, their investigation was currently limited to the behavioral level, and the available evidence was insufficient. Therefore, the current study aims to employ both behavioral and neural methods (electroencephalography, EEG) to test our hypotheses and propose a specific time course.
Research sample	We conducted an a priori power analysis using G*Power 3.1 (Faul et al., 2007). With a desired power of 0.80, a significance level of 0.05, and an effect size of f = 0.25, the power analysis suggested an estimated sample size of approximately 24 participants. Thirty-three healthy participants, including 20 females and 13 males with a mean age of 20.84 ± 2.18 years (range: 18-29 years) from local city participated in this experiment. All participants were right-handed and had normal or corrected-to-normal vision. All participants completed a questionnaire regarding their gender identity, with a free-response option to indicate male, female, or other.
Sampling strategy	We conducted an a priori power analysis using G*Power 3.1 (Faul et al., 2007). With a desired power of 0.80, a significance level of 0.05, and an effect size of f = 0.25, the power analysis suggested an estimated sample size of approximately 24 participants. Random
Data collection	The experimental procedure was conducted on a 19-inch monitor (1280 x 1024 pixels, 60 Hz refresh rate), with stimulus presentation and data acquisition performed using E-Prime software 2.0.10. EEG data were collected using standard 64 in-cap Ag/AgCl electrodes following the extended international 10 – 20 system (Brain Products).
Timing	2022.11.28 to 2022.12.15
Data exclusions	No data were excluded from analysis
Non-participation	no participants dropped out
Randomization	This study employs a within-subjects design without any groupings.

Reporting for specific materials, systems and methods

We require information from authors about some types of materials, experimental systems and methods used in many studies. Here, indicate whether each material, system or method listed is relevant to your study. If you are not sure if a list item applies to your research, read the appropriate section before selecting a response.

Materials & experimental systems

n/a	Involved in the study
☒	☐ Antibodies
☒	☐ Eukaryotic cell lines
☒	☐ Palaeontology and archaeology
☒	☐ Animals and other organisms
☒	☐ Clinical data
☒	☐ Dual use research of concern
☒	☐ Plants

Methods

n/a	Involved in the study
☒	☐ ChIP-seq
☒	☐ Flow cytometry
☒	☐ MRI-based neuroimaging

Plants

Seed stocks	no seed stocks
Novel plant genotypes	no novel plant genotypes
Authentication	no authentication
